# Factors affecting optimal adherence to antiretroviral therapy and viral suppression amongst HIV-infected prisoners in South Ethiopia: a comparative cross-sectional study

**DOI:** 10.1186/s12981-022-00429-4

**Published:** 2022-01-29

**Authors:** Terefe Gone Fuge, George Tsourtos, Emma R. Miller

**Affiliations:** College of Medicine and Public Health, Flinders University, Adelaide, Australia

**Keywords:** Incarceration, Antiretroviral therapy, Adherence, Viral suppression, South Ethiopia

## Abstract

**Background:**

Maintaining optimal adherence and viral suppression in people living with HIV (PLWH) is essential to ensure both preventative and therapeutic benefits of antiretroviral therapy (ART). Prisoners bear a particularly high burden of HIV infection and are highly likely to transmit to others during and after incarceration. However, the level of treatment adherence and viral suppression in incarcerated populations in low-income countries is unknown. This study aimed to determine factors affecting optimal adherence to antiretroviral therapy and viral suppression amongst HIV-infected prisoners in South Ethiopia.

**Methods:**

A comparative cross-sectional study was conducted between June 1, 2019 and May 31, 2020 to compare the level of adherence and viral suppression between incarcerated and non-incarcerated PLWH. Patient information including demographic, socio-economic, behavioral, and incarceration-related characteristics were collected using a structured questionnaire. Medication adherence was assessed according to self-report and pharmacy refill. Plasma viral load measurements undertaken within the study period were prospectively extracted to determine viral suppression. Univariate and multivariate logistic and fractional regression models were used to analyse data.

**Results:**

Seventy-four inmates living with HIV (ILWH) and 296 non-incarcerated PLWH participated in the study. While ILWH had a significantly higher pharmacy refill adherence compared to non-incarcerated PLWH (89 vs 75%), they had a slightly lower dose adherence (81% vs 83%). The prevalence of viral non-suppression was also slightly higher in ILWH (6.0%; 95% confidence interval (CI): 1.7–14.6%) compared to non-incarcerated PLWH (4.5%; 95%CI: 2.4–7.5%). Overall, missing ART appointments, dissatisfaction with ART services, inability to comply with a specified medication schedule, and types of methods used to monitor the schedule (e.g., news time on radio/TV or other social cues) were significantly associated with non-adherence according to self-report. In ILWH specifically, accessing ART services from a hospital, inability to properly attend clinic appointments, depressive symptoms, and lack of social support predicted NA. Viral non-suppression was significantly higher in males, people of age 31to 35 years and in those who experienced social stigma, regardless of their incarceration status.

**Conclusions:**

Sub-optimal dose adherence and viral suppression are generally higher in HIV-infected prisoners in South Ethiopia compared to their non-incarcerated counterparts. A multitude of factors were found to be responsible for this requiring multilevel intervention strategies focusing on the specific needs of prisoners.

## Background

Although there has been a steep decline in the number of new HIV infections and associated deaths in the general population worldwide, key populations such as prisoners remain disproportionately affected by the epidemic, accounting for more than half of all new infections [1]. There is considerably higher HIV prevalence in the prisons of sub-Saharan Africa (SSA), reaching up to 35% in some countries [2]. An HIV prevalence of greater than 4% has been documented in Ethiopian prisons [3], which is more than four times higher than the prevalence in the general Ethiopian population, and one of the highest HIV prevalences in prison populations in SSA relative to the general population [1].

Antiretroviral therapy (ART) is associated with significantly reduced HIV-associated morbidities and mortality. ART is also believed to prevent HIV transmission by suppressing viral load in infected individuals [4–6]. To ensure the public health benefits, people living with HIV (PLWH) need to have optimal treatment adherence and achieve viral suppression [7]. Given the overall high prevalence of HIV in prisoners, most of whom will reintegrate into the general community, poor treatment outcomes in such populations may facilitate onward transmission [1, 8].

Promising outcomes have been reported amongst prisoners in both high- and low-income countries (including those in SSA) regarding ART adherence and viral suppression in prisons where standard HIV care is implemented [9–12]. However, the prevalence of non-adherence (NA) and viral non-suppression remains high in prisons of many countries, with more than half of inmates living with HIV (ILWH) having sub-optimal treatment outcomes in some settings [13–15]. Several institutional, psychosocial and personal factors have previously been reported to affect optimal adherence and viral suppression in prisoners. Cooperativeness of security systems [16–18], type of ART service delivery approach (for example, provision of ART via directly observed therapy (DOT) or accessing care from external ART sites) [18, 19], the nature of inmate-health care provider relationships [19, 20], and food supply insufficiency (particularly in resource limited countries) [18, 21, 22] have been reported to be the main institutional circumstances to affect ART outcomes.

Psychosocial factors such as social support, stigma and depression have been found to influence ART adherence in prisoners. It has been shown that ILWH who are able to receive social support (be it material, emotional or information support), either from inside or outside of a prison, are more likely to be adherent to ART than those who are not [13, 14, 20]. In contrast, social stigma perpetuated by prison staff and fellow inmates negatively affects adherence [17–19]. Further, ILWH often have a high prevalence of depression [23] which may have substantial adverse effects on their ART adherence and viral suppression [9, 13, 15].

With only limited data existing regarding personal factors affecting ART adherence and viral suppression in prisoners, it has been reported that self-perceptions of HIV status, the health benefits of ART as well as its potential adverse consequences have been associated with NA. For instance, ILWH who perceive that ART is inefficient and has side-effects are less likely to adhere to ART [14, 24]. The odds of NA is higher in ILWH who have experienced more frequent antiretroviral (ARV) side-effects [15, 21, 25] and other underlying disease symptoms [14, 26]; and non-adherent ILWH are, in turn, less likely to achieve viral suppression [14]. Having a history of injecting drug use is the only major behavioural factor that has been statistically confirmed to be negatively associated with ART adherence and viral suppression in ILWH [14, 26, 27]. Other personal characteristics reported to be associated with NA and viral non-suppression are younger age (below 35 years) and being male [9, 22, 27, 28].

There have been reports showing that ILWH have limited access to HIV care in many SSA prisons [2, 18, 29], however it remains unclear to what extent incarceration affects optimal ART adherence and viral suppression in such settings. No published studies have quantitatively investigated ART outcomes in the prison systems of Ethiopia previously, although there have been suggestions that population groups commonly referred to as ‘Most at Risk Groups’ (MARPS) for HIV (including prisoners) remain with restricted access to care [30]. This study therefore aimed to determine factors affecting optimal adherence to antiretroviral therapy and viral suppression amongst HIV-infected prisoners in South Ethiopia relative to their non-incarcerated counterparts.

## Methods

### Study design and setting

A comparative cross-sectional study was conducted between June 1, 2019 and May 31, 2020 to compare the outcomes of ART between HIV-infected incarcerated and non-incarcerated individuals in South Ethiopia. We have provided a detailed description of the study setting elsewhere [31]. In brief, approximately one quarter of the correctional facilities (six of 23 prisons) in South Ethiopia and public health care facilities offering ART services for the prisoners were involved in the study. Prisons with a high load of inmates were chosen purposively to obtain adequate number of prisoners that bear socio-cultural diversity. The prisons are located in the central part of Ethiopia and accommodate people originating from diverse areas of the region and the country, including rural areas.

### Participant recruitment

**Fig. 1:**
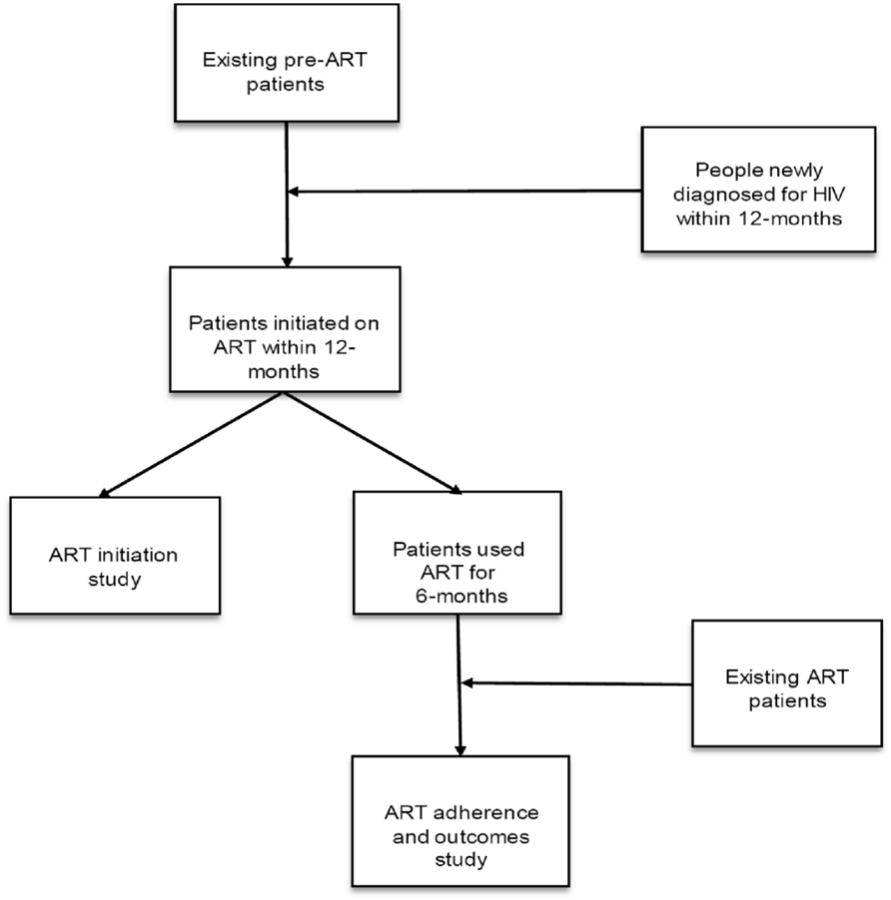
Participant recruitment process

All HIV-infected prisoners who were on ART during the study enrolment period were eligible for participation as a risk group. The comparison groups included HIV-infected non-incarcerated people who were receiving care from the same ART clinics and had similar ART history as the prisoners. Patients who had initiated ART before the beginning of the study and new patients who started ART within the first six months of the study were included. Figure 1 shows the process of participant recruitment. As the population pool for non-incarcerated people was much larger than incarcerated people, a simple random sampling technique [32] was used to recruit a sample of non-incarcerated participants that quadrupled the number of prisoner participants. To assist in this, a list of clients in ART registers served as a sampling frame to select potential participants using a table of random numbers.

#### Inclusion criteria

Study participants included HIV-infected persons who were able to provide written informed consent, aged 18 years and above, and were receiving HIV care for at least six months at one of the selected ART clinics in South Ethiopia. Six or more months adherence measurement was used in order to rule out a particular risk of poor adherence during the earlier months of ART [33].

#### Exclusion criteria

Individuals who were seriously unwell (as determined by ART service providers) and unable to provide complete information were excluded. Non-incarcerated PLWH with a previous history of imprisonment and ILWH who had already developed non-adherence and/or had viral non-suppression before incarceration (as confirmed through clinical chart reviews) were also excluded. ILWH were also required to remain imprisoned for at least one month.

### Sample size determination

The smallest difference in the proportion of NA between incarcerated and non-incarcerated people was considered to determine the minimum sample size required to identify an estimated prevalence ratio. A formula for unmatched cross-sectional studies [32]; assuming 95% of level of confidence, 80% power, a 5% level of significance and unexposed to exposed group sample ratio of four was used to calculate the sample size. Considering a proportion of 24.4% NA in the general population in Ethiopia [34], and a prevalence ratio of 1.67 in NA in the incarcerated population [22], a minimum sample of 74 inmates was required. As four times the number of incarcerated participants was required compared to the non-incarcerated group, a final sample size of 370 participants was recruited from both populations.

#### Data collection procedure

ART service providers at the participating public health care facilities invited potential participants to see a trained research assistant in a separate room. The invitation occurred when PLWH made their regular clinic visit. The research assistants were certified HIV counsellors who had a tertiary qualification in health-related disciplines. Participants underwent Paper and Pencil Interviewing (PAPI) about their background information and self-reported adherence to medication once they gave consent for participation to the research assistant.

To minimise the effect of the language barrier on the accuracy of PAPI data, the questionnaire, which was initially prepared in English language, was translated into Amharic, a commonly spoken language in the study area. Completed questionnaires were then translated back into English at the end of the data collection process. Pre-testing was conducted to ensure context validity (i.e. clarity, meaningfulness and difficulty) of questionnaire items with a group of participants representing five percent of the study sample size; using incarcerated and non-incarcerated PLWH at ART clinics remote to the study sites. As lay experts [35], one ART service provider from each study health facility evaluated the face validity of the questionnaire. To perform this, the ART service providers were provided with the questionnaire ahead of the data collection process. Although some items of the questionnaire were obtained from previously validated instruments (as described below), newly developed items were tested for internal consistency using Cronbach’s α [36], and corrections were made by removing less consistent items based on the ‘α’ values of the pre-test data.

### Variables and measurements

#### Background information

In the questionnaire, participants were asked about their sociodemographic, psychosocial (social support, stigma and depression), behavioural, and incarceration related characteristics. The core components of social support including emotional, informational, tangible, comradeship and positive social interactions [37] were assessed using nine items, part of which were adapted from a multi-item scale developed by White et al. [24], which had internal consistency (α) of 0.79. The items were further checked for contextual reliability and showed an acceptable Cronbach’s α value (α = 0.66). The four manifestations of HIV-related social stigma: internalised (negative self-image), enacted (personalised), perceived (concern with public attitude) and concerns with status disclosure [38] were measured using a shortened version [39] of the 40-item scale by Berger et al. [38] (α > 0.7). Non-specific psychological distress was assessed using a six-item scale developed by Kessler et al.[40] (α = 0.89). Participant responses were graded using a five-point Likert scale ranging from 1, “Strongly disagree” to 5, “Strongly agree” for social support and stigma measurements, and a four-category scale (most of the time, some of the time, a little of the time, none of the time) for depression.

Knowledge and attitudes of HIV and ART, as well as self-efficacy in medication use were assessed using items generated from the literature review. The knowledge scale consisted of eight items, and the attitude and self-efficacy scales each consisted of three-item questions. The scales showed sufficient Cronbach’s α values; 0.78 and 0.65 for the knowledge and attitude constructs, respectively. Whereas responses for the knowledge items were scored by assigning one point for every correct response and zero for an incorrect answer, a five-point response scale was used for the attitude and self-efficacy items. In each measurement scale, scores were summed to determine the overall score, and the interquartile range was calculated to categorise results.

#### Adherence to ART

Adherence was measured using the participants’ self-report and pharmacy refill records to offset the limitations of one method by the other [41–43]. Participants were asked once to self-report their medication use in the previous four days, an ideal time interval to minimise possible recall and social desirability bias [43]. The percentage of adherence was then determined by calculating the proportion of pills taken of the number of pills prescribed:

$$ Self - reported\, adherence = \frac{number\, of\, pills\, taken\, in\, the\, last\, four\, days }{{number\, of\, pills\, prescribed\, for\, the\, last\, four\, days }}x100 $$

Participants were also assessed once for their six-monthly (180 days) pharmacy refill adherence to any prescribed ARV drugs. Variation in the medication possession ratio (MPR) was determined by dividing the number of days a patient was late for pharmacy refills by the total days on ART regardless of left-over medications, and then subtracting this proportion from 100% [44, 45]. i.e.

$$ MPR = 1 - \frac{number\, of\, days\, late\, for\, ARV\, pick - up}{{Total\, number\, of\, days\, between\, the\, two\, most\, recent\, ARV\, pick - ups}}x100 $$

For both self-report and pharmacy refill methods, patients with an adherence percentage of < 95% were considered as non-adherent [46, 47]. Participants were also asked to self-report on their adherence to dose schedules and medication instructions in the previous four days or more and complete a brief survey on potential risk factors for NA.

#### Viral suppression

The South Ethiopian Regional Public Health Laboratory (RPHL) performs viral load tests using plasma samples for HIV patients six months from ART initiation and every 12 months thereafter. Of investigations undertaken within 12 months of the study period, the most recent ones were prospectively extracted from the laboratory registers using patient medication identification numbers. Although the lowest detection limit of the testing machine was 40 copies/mL, viral non-suppression in this study was defined as viral load above 1000 copies/mL, which is partly adapted from World Health Organization (WHO) definitions [42].

### Data analysis

Data were manually checked for completeness, consistency and cleanness, entered into an EpiData (version 4.6) template, and then exported to Stata (StataCorp. 2019. Stata Statistical Software: Release 16. College Station, TX: StataCorp LLC.) for analysis [48]. Participant characteristics were initially described in terms of frequencies and percentages for categorical variables, while summary statistics such as means, medians, standard deviations and quartiles were calculated for continuous variables. Bivariate associations amongst categorical variables were estimated using Chi-square (Chi^2^) test [49] and mean and median differences in continuous variables between the target and comparison populations were determined using T-test and Mann–Whitney U-test respectively [50].

Adherence was analysed in two ways: as a continuous outcome restricted to the interval between 0 and 1, and a binary outcome categorised as adherence and non-adherence (NA). In the first case, as the data included the upper and lower bounds [0, 1], a fractional regression model was used to estimate the results with logit as a link function [51]. Covariates of NA and viral non-suppression were determined using a logistic regression model [52].

The models were developed through a purposeful selection approach [52]. Each variable was initially independently tested using a bivariate regression model to identify eligible variables for a multivariate regression model. A relatively less stringent inclusion criterion (*P* < 0.2) was used to ensure the consideration of all potentially important covariates in the analyses. Variables which did not contribute to the model at the traditional significance level (*P* < 0.05) as well as those which did not appear to have a significant confounding effect (_β_ < 20%) were eliminated [52].

Interaction terms were considered when they were found to have a statistically significant effect [53]. Multicollinearity between continuous covariates was tested thorough scatterplots and a bivariate linear regression, whereas a correlation coefficient as well as a variance inflation factor was determined for both continuous and categorical variables [53]. All the covariates included in the models had a variance inflation factor value of less than 1.5.

A significance of associations between covariates and the outcome variables was determined at a *P* < 0.05 with 95% confidence interval (CI). Goodness-of-fit for the logistic regression models was assessed using Hosmer and Lemeshow Chi^2^ test [52] whereas that of the fractional regression analyses was checked using a generalised linear model applying logit as a link function [54, 55]. In all cases, the models fit the data well.

Missing values were observed within several dependent and independent variables in the dataset. For variables included in the analysis models, Little’s test was used to check whether the missingness occurred completely at random (MCAR), independent of observed and unobserved values [56]. The test identified that the pattern of missingness varied across the variables and violated the assumption of MCAR. Thus, the multiple imputation technique (*m* = 20) was applied to take into account the effect of missing values, in which the results obtained from each completed-data analysis were combined to produce a single multiple-imputation result. The fit of the imputation models was checked using a graphical method [57]. The distribution of the observed and completed values appeared to be comparable.

## Results

### Participant characteristics

One hundred and twenty-two ILWH were identified in the six selected correctional facilities. Of these, 24 (19.7%) ILWH did not participate due to their release from prison ahead of their clinic appointment at which the consenting process would have been conducted. Ten ILWH (8.2%) failed to meet the study eligibility criteria. Of the remaining 88 ILWH, 74 agreed to participate in the study, which gives a response rate of 84.1%. During the study period, there was a total of 3806 non-incarcerated adult PLWH who were receiving ART services at the six selected public health care facilities; of whom, 296 were randomly selected for participation in this study as comparators.

Characteristics of participants are described in Table 1. The majority 66 (89%) of ILWH participants were male, as were only 139 (47%) non-incarcerated PLWH participants. Both groups were of comparable age; the median age of ILWH was 34 years (Interquartile range (IQR): 28–40 years) whereas that of non-incarcerated PLWH was 35 years (IQR: 30–40 years). Ninety-eight (33%) non-incarcerated PLWH participants reported having completed high school and above education whereas 13 (18%) ILWH participants reported attaining similar educational level (P = 0.015). ILWH were more likely to be farmers or daily labourers prior to incarceration. Most 67 (23%) non-incarcerated PLWH were housewife. Half of the ILWH reported urban areas as their last residence before incarceration whereas 204 (69%) non-incarcerated PLWH were urban residents (P = 0.013). Thirteen (18%) ILWH reported experiencing a homelessness.

**Table 1:** Characteristics of incarcerated and non-incarcerated people living with HIV in South Ethiopia (N = 370)

Characteristic		Incarcerated (N = 74), n (%)	Non-incarcerated (N = 296), n (%)	P-value (Chi^2^)
Gender	Male	66 (89.2)	139 (47.0)	0.000
	Female	8 (10.8)	157 (53.0)	
Age in years	18–25	13 (17.6)	30 (10.1)	0.157
	26–30	18 (24.3)	62 (21.9)	
	31–35	14 (18.9)	57 (19.3)	
	> 35	29 (39.2)	147 (49.7)	
Current marital status	Have partner	35 (47.3)	169 (57.1)	0.067
	Have no partner	39 (52.7)	127 (42.9)	
Highest level of education completed	No school	24 (32.4)	82 (27.7)	0.015
	Elementary school	37 (50.0)	116 (39.2)	
	High school	11(14.9)	66 (22.3)	
	College graduate	2 (2.7)	32 (10.8)	
Employment status				0.000
	Unemployed	4 (5.4)	19 (6.4)	
	Government employee	7 (9.4)	60 (20.3)	
	Home duties	2 (2.7)	67 (22.6)	
	Farmer	19 (25.7)	46 (15.5)	
	Daily labourer	19 (25.7)	55 (18.6)	
	Other	23 (31.1)	49 (16.6)	
Monthly income in USD	≤ 13.5	22 (31.0)	110 (37.4)	0.480
	13.6–22.8	8 (11.3)	24 (8.2)	
	> 22.8	41 (57.7)	160 (54.4)	
Residence	Urban	37 (50.0)	204 (68.9)	0.013
	Rural	27 (36.5)	68 (23.0)	
	Both	10 (13.5)	23 (7.8)	
	Unknown	0 (0.0)	1 (0.3)	
History of homelessness	No	61 (82.4)	284 (96.0)	0.000
	Yes	13 (17.6)	12 (4.0)	
Length of current incarceration in months	< 12	28 (37.8)	–	–
	≥ 12	46 (62.2)	–	
Length of current sentence in months	< 12	2 (3.0)	–	–
	12–59	24 (36.4)	–	–
	60–119	16 (24.2)	–	–
	≥ 120	24 (36.4)	–	–
Number of incarcerations	1	61 (82.4)	–	–
	> 1	13 (17.6)	–	
Dissatisfaction with ART services	_	65 (87.7)	234 (79.1)	0.024

*ART* antiretroviral therapy, *USD* United States dollars

^*^Residence, employment status and monthly income for incarcerated people refer to the last circumstances before incarceration

^*^Sum of ‘monthly income’ and ‘length of current sentence’ categories may not give the total sample due to missing data (1.3% and 10.5% respectively)

### Non-adherence to ART

The median duration of ART use was 44 months (IQR: 24–68 months) for prisoners and 48 months (IQR: 23–72 months) for non-incarcerated clients. Sixty-four (17%) participants had non-adherence (NA) by self-report and 82 (22%) by pharmacy refill methods. While prisoners had a significantly lower pharmacy refill non-adherence (11%) compared to non-incarcerated clients (15%) (P = 0.009), they had a slightly higher dose non-adherence (see Table 2).

**Table 2:** Clinical characteristics of incarcerated and non-incarcerated people living with HIV in South Ethiopia (N = 370)

Characteristic		Incarcerated, n (%)	Non-incarcerated, n (%)	P-value (Chi^2^)
ART adherence	Non-adherence by self-report	14 (18.9)	50 (16.9)	0.680
	Non-adherence by pharmacy refill	8 (10.8)	74 (25.0)	0.009
Viral suppression	Viral non-suppression	4 (6.0)	13 (4.4)	0.598

*Sum of ‘viral suppression’ categories may not give the total sample due to missing data (3%).

### Viral non-suppression

Seventeen (4.7%) participants were found to have viral non-suppression. Prisoners had a slightly higher viral non-suppression compared to non-incarcerated people—four out of 67 (6%) ILWH participants had viral non-suppression (see Table 2).

### Factors associated with ART non-adherence

Various factors were identified as determinants of overall dose-NA in a multivariate logistic regression analysis (see Table 3). The analysis indicated that missing a clinic appointment increases the odds of dose-NA. For instance, the odds of being adherent to ART decreased by 94% among patients who missed at least one clinic appointment compared to patients who didn't miss their clinic appointment (Adjusted odds ratio (AOR): 0.06; 95%CI: 0.02–0.22). The ability to strictly adhere to a specific medication schedule was a determinant of dose adherence in incarcerated and non-incarcerated clients. Accordingly, the odds of dose adherence was 99% lower in those who were able to keep their medication schedule most of the time rather than all of the time (AOR: 0.01; 95%CI: 0.002–0.13) and 99.8% lower in those who never followed their medication schedule (AOR: 0.002; 95%CI: 0.0001–0.05). Methods that participants used to manage their medication schedule also appeared to affect dose adherence. Clients who used news time on radio/TV or other social cues, such as sunlight or departure time to school/church/mosque were less likely to comply with doses relative to those who were able to use one or more time monitoring devices such as mobile phones, wristwatches, etc. (AOR: 0.08; 95%CI: 0.01–0.53 vs AOR: 0.07; 95%CI: 0.01–0.67). In addition, the risk of dose-NA was more than seven times higher in clients who had poor satisfaction with ART services (AOR: 0.14; 95%CI: 0.03–0.63), which was higher in incarcerated ART clients than their non-incarcerated counterparts (see Table 3).

**Table 3:** Logistic regression model of factors associated with self-reported ART non-adherence amongst incarcerated and non-incarcerated ART clients in South Ethiopia (incarcerated = 74; non-incarcerated = 296)

Variable		Adherence		COR (95% CI)	AOR (95% CI)
		Adherent, n (%)	Non-adherent, n (%)		
Relationship with a person to whom HIV status disclosed	Spouse	48 (92.3)	4 (7.7)	3.07 (1.03–9.12)*	1.71 (0.38–7.71)
	Offspring	11 (64.7)	6 (35.3)	0.47 (0.16–1.36)	0.58 (0.11–3.02)
	Parent	10 (71.4)	4 (28.6)	0.64(0.19–2.17)	0.98 (0.13–7.23)
	More than one of the above	129 (79.6)	33 (20.4)	1	1
Adherence to specific medication schedule in the last four days	Never	6 (75.0)	2 (25.0)	0.08 (0.01–0.51)*	0.002 (0.0001–0.05)*
	Some	6 (54.5)	5 (45.5)	0.03 (0.01–0.15)*	0.03 (0.002–0.52)*
	Half	20 (57.1)	15 (42.9)	0.04 (0.01–0.12)*	0.002 (0.0001–0.02)*
	Most	120 (75.9)	38 (24.1)	0.08 (0.03–0.24)*	0.01(0.002–0.13)*
	All of the time	154 (97.5)	4 (2.5)	1	1
Aids used to manage medication schedule	Mobile phone	33 (80.5)	8 (19.5)	0.67 (0.28–1.61)	0.35 (0.07–1.72)
	Watch	24 (72.7)	9 (27.3)	0.43 (0.18–1.03)	0.23 (0.04–1.26)
	Radio/TV	19 (63.3)	11(36.7)	0.28 (0.12–0.66)*	0.08 (0.01–0.53)*
	Other	26 (74.3)	9 (25.7)	0.47 (0.20–1.11)	0.07 (0.01–0.67)*
	More than one aid	166 (86.0)	27 (14.0)	1	1
Number of clinic appointments missed in the last 12-months	None	210 (93.8)	14 (6.3)	1	1
	One	83 (65.3)	44 (34.7)	0.13 (0.07–0.24)*	0.06 (0.02–0.22)*
	Two and more	13 (68.4)	6 (31.6)	0.14 (0.05–0.44)*	0.16 (0.03–0.97)*
Satisfaction with ART services	Poor	242 (80.9)	57 (19.1)	0.46 (0.20–1.06)	0.14 (0.03–0.63)*
	Good	64 (90.1)	7 (9.9)	1	1

*COR* crude odds ratio, *AOR* adjusted odds ratio, *CI* confidence interval, *ART* antiretroviral therapy, *TV* television; ^*^ statistically significant association at *P* < 0.05.

Adherent: adherence level ≥ 95%; Non-adherent: adherence level < 95%.

Sum of categories of ‘relationship with a person to whom HIV status disclosed’ and ‘aids used to manage medication schedule’ may not give the total sample as some categories were not considered in the analysis due to an insufficient number of observations.

We specifically assessed predictors of non-adherence to doses and pharmacy refill in prisoners using a multivariate fractional regression analysis (see Tables 4 and 5). Prisoners who were accessing ART services from a hospital were 75% less likely to comply with scheduled doses (AOR: 0.25; 95%CI: 0.07–0.90) compared to prisoners who were accessing the services from a health centre. The risk of dose-NA increased by 93% when prisoners missed a single ART appointment (AOR: 0.07; 95%CI: 0.01–0.67) and by 99% when they missed two or more appointments (AOR: 0.01; 95%CI: 0.002–0.08). Inmates with depressive symptoms had a 74% lower likelihood of dose adherence than those without depressive symptoms (AOR: 0.26; 95%CI: 0.07–0.88) (see Table 4).

**Table 4:** Fractional regression model of factors associated with self-reported dose adherence amongst incarcerated people living with HIV in South Ethiopia (N = 74)

Variable		COR (95% CI)	AOR (95% CI)
Employment status	Unemployed	1.20 (0.07–20.00)	0.09 (0.004–2.03)
	Government employee	1	1
	Farmer	3.40 (0.37–31.12)	2.46 (0.81–7.44)
	Daily labourer	0.867 (0.13–5.89)	0.69 (0.28–1.72)
	Others	2.67 (0.34–20.79)	0.70 (0.34–1.44)
Having depressive symptoms	No	1	1
	Yes	0.28 (0.08–0.95)*	0.26 (0.07–0.88)*
Type of health facility	Health centre	1	1
	Hospital	0.89 (0.26–3.02)	0.25 (0.07–0.90)*
Number of clinic appointments missed in the last 12-months	None	1	1
	1	0.04 (0.01–0.19)*	0.07 (0.01–0.67)*
	≥ 2	0.02 (0.001–0.33)*	0.01 (0.002–0.08)*

*COR* crude odds ratio, *AOR* adjusted odds ratio, *CI* confidence interval; *ART* antiretroviral therapy; * statistically significant association at *P* < 0.05.

Employment status refers to the last occupation before incarceration.

Sum of categories of ‘employment status’ may not give the total sample as some categories were not considered in the analysis due to an insufficient number of observations.

Similar to dose adherence, accessing ART services from a hospital decreased the inmates’ pharmacy refill adherence by 95% compared to accessing the services from a health centre (AOR: 0.05; 95%CI: 0.02–0.13). Prisoners who had viral non-suppression were more than two times less likely to comply with pharmacy refill (AOR: 0.38; 95%CI: 0.20–0.73). Moreover, the likelihood of pharmacy refill adherence was 86% lower in inmates who reported lacking ILWH-roommates (AOR: 0.14; 95%CI: 0.05–0.40) (see Table 5).

**Table 5:** Fractional regression model of factors associated with pharmacy refill adherence amongst incarcerated people living with HIV in South Ethiopia (N = 74)

Variable		COR (95% CI)	AOR (95% CI)
Length of time on ART in months	–	1.01 (1.001–1.02)*	1.01 (0.99–1.03)
ART use before incarceration	No	2.54 (0.88–7.35)	2.78 (0.99–7.79)
	Yes	1	1
Presence of anyone living with HIV in a cell	No	0.26 (0.08–0.79)*	0.14 (0.05–0.40)*
	Yes	1	1
Type of health facility	Health centre	1	1
	Hospital	0.28 (0.10–0.82)*	0.05 (0.02–0.13)*
Viral non-suppression ^m^	No	1	1
	Yes	0.12 (0.02–0.64)*	0.38 (0.20–0.73)*

*COR* crude odds ratio, *AOR* adjusted odds ratio, *CI* confidence interval, *ART* antiretroviral therapy, *MPR* medication possession ratio (pharmacy refill); ^m^ variable with missing value; ^*^ statistically significant association at *P* < 0.05; –: not applicable.

The effect of missingness in this particular dataset is negligible as the complete case analysis and multiple imputation gave exactly the same AORs (results not displayed).

### Factors associated with viral non-suppression

A multivariate logistic regression identified predictors of overall viral non-suppression in incarcerated and non-incarcerated ART clients. The estimation was made based on a complete case analysis and multiple imputation of variables with missing values (see Table 6). Sociodemographic factors such as gender, age and social stigma appeared to be determinants of viral non-suppression in both analyses. In the complete case analysis, the risk of viral non-suppression was 97% higher in males than females (AOR: 0.03; 95%CI: 0.003–0.41) whereas 96% higher in multiple imputation (AOR: 0.04; 95%CI: 0.003–0.41). ART clients in the age group of 31 to 35 years had more than fourteen times the risk of having viral non-suppression relative to those who were > 35 years old in the complete case analysis (AOR: 14.10; 95%CI: 2.35–84.57) and about thirteen times higher risk in the multiple imputation (AOR: 13.05; 95%CI: 2.10–81.16). Experiencing social stigma increased the risk of viral non-suppression more than tenfold both in the complete case analysis and multiple imputation (AOR: 10.59; 95%CI: 1.81–62.03 vs AOR: 10.19; 95%CI: 1.77–58.57).

**Table 6:** Logistic regression model of factors associated with virological failure amongst incarcerated and non-incarcerated ART clients in South Ethiopia

Variable		Virological failure		COR (95% CI)	AOR (95% CI), Complete case analysis (N = 279)	AOR (95% CI), Multiple imputation (N = 370)
		Yes, n (%)	No, n (%)			
Gender	Male	16 (8.0)	183 (92.0)	1	1	1
	Female	1 (0.4)	156 (99.4)	0.07 (0.01–0.55)*	0.03 (0.003–0.41)*	0.04 (0.003–0.41)*
Age in years	26–30	3 (3.9)	74 (96.1)	1.12 (0.27–4.61)	5.37 (0.62–46.34)	4.89 (0.54–44.32)
	31–35	8 (11.4)	62 (88.6)	3.57 (1.19–10.70)*	14.10 (2.35–84.57)*	13.05 (2.10–81.16)*
	> 35	6 (3.5)	166 (96.5)	1	1	1
Social stigma	Non-stigmatised	9 (3.3)	262 (96.7)	1	1	1
	Stigmatised	8 (9.1)	80 (90.9)	2.91 (1.09–7.79)*	10.59 (1.81–62.03)*	10.19 (1.77–58.57)*
Follow up CD4 count^m^	–	–	–	1.00 (0.99–1.00)	0.998 (0.995–1.000)	0.998 (0.995–1.00)

*COR* crude odds ratio, *AOR* adjusted odds ratio *CI* confidence interval, *ART* antiretroviral therapy; ^m^ variable with missing value; ^*^ statistically significant association at *P* < 0.05; –: not applicable.

Sum of the age categories may not give the total sample as a category ‘18–25 years’ was not considered in the analysis due to an insufficient number of observations.

## Discussion

This study aimed to identify factors affecting optimal adherence to ART and viral suppression in prisoners in South Ethiopia. Prisoners had a level of viral suppression (94%) which is close to the third goal of the Joint United Nations Programme on HIV/AIDS (UNAIDS), i.e. achieving viral suppression in 95% of treated individuals by 2030 [58]. They also had a lower prevalence of non-adherence (NA) and viral non-suppression than that commonly reported in SSA general populations [59, 60] including that of Ethiopia [33, 61], as well as in prison populations internationally [9, 10, 14, 15, 62]. However, NA and viral non-suppression prevalence amongst prisoners in this study was higher relative to the local non-incarcerated population. The recent and rapid expansion of ART services in Ethiopia might have contributed to the positive treatment outcomes in this study [7, 63], but the discrepancy between incarcerated and non-incarcerated individuals may suggest an inequitable access to standard HIV care between community- and correctional facility-based populations. The findings also indicate the importance of a patient’s compliance with specified doses for achieving viral suppression [64, 65], which predicts HIV-related morbidities and mortality, as well as further transmission [4–6].

Various structural, psychosocial, individual and clinical factors were identified to influence ART adherence and viral suppression in ILWH relative to non-incarcerated PLWH. While missing ART appointments was an important factor affecting adherence in both incarcerated and non-incarcerated populations, it appeared to be more critical in ILWH. Regular clinic visits are essential for ART clients in order to receive ongoing adherence counselling and support services, as well as clinical assessment and further prescription of ART [42]. Omission of such appointments, therefore, subsequently leads to sub-optimal adherence and facilitation of community transmission [8, 42, 47, 66]. Prisoners in low-income countries often access ART services from external public health care facilities, which presents serious of institutional barriers (e.g. a lack of transport facilities and uncooperative security system) [18, 67]. We recommend implementation of standard HIV care package in the prison system as supported by international guidelines [23, 68, 69].

A significantly lower level of satisfaction with ART services was observed in ILWH than non-incarcerated PLWH. This is important because the results revealed an 86% lower likelihood of dose adherence in clients who had low satisfaction. Furthermore, the odds of dose adherence were 75% lower in ILWH who had received ART services from a hospital compared to those who were receiving ART services from a health centre. The importance of good health care provider-patient relationships for enhancing adherence is well recognised both in prison [19, 20, 26] and community-based populations [70, 71]. However, health care provider- and health facility-related issues (e.g., long waiting time) are amongst the most frequently reported barriers to ART adherence in SSA [72]. The findings therefore suggest a need for decentralisation of ART services to primary health care facilities including prison clinics. Training of health care providers in HIV care provision is pivotal to achieve this, in addition to reinforcing collaboration between prison and community healthcare systems [73].

Our study showed a significant decrease in the odds of adherence in prisoners with depressive symptoms and in those who lacked social support. Although depression strongly predicts NA in the community-based populations as well [74–76], ILWH often feel depressed due to concerns related to imprisonment [23] and HIV infection itself [77]. The positive impact of social support on prisoners’ ART adherence and the likely increase in the risk of NA when ILWH suffer from social isolation is well recognised [14, 20, 78]. Thus, in addition to enhancing peer support programs in prison settings, integration of HIV care and treatment of medically diagnosed depression is likely to be essential for maintaining ART adherence in prisoners.

Among the individual level factors assessed in this study, the ability to comply with a specified medication schedule determined dose adherence in incarcerated and non-incarcerated ART clients. Our study also signified that the type of methods clients used to manage their medication schedule affected dose adherence. For example, dose adherence significantly decreased in clients who used news time on radio/TV or other social cues compared to those who used more direct methods, such as mobile phones and/or wristwatches. Research shows that patients’ ability to comply with medication instructions generally increases when they perceive good efficacy and safety of ART [14, 24, 71]. In addition, the use of reminder devices such as telephone reminders, clocks and alarms has been associated with a significant increase in ART adherence [79–81]. Adapting such interventions to prison context and the specific needs of prisoners is required.

In the current study, ILWH who experienced viral non-suppression had a significantly lower MPR adherence. Prior studies have shown that having NA lessens the likelihood of viral suppression in both prison- [14] and community-based populations [34, 82–84]. However, the current study provided no evidence regarding such a relationship, which might be due to the small number of participants who had developed the clinical outcomes. Nonetheless, people with a higher plasma viral load [85–87] and other disease symptoms [14, 26] often find it challenging to consistently use their medication. This could be related to a high pill burden and potential drug interactions that are likely to occur during the advanced stages of HIV infection due to opportunistic infections [59, 88, 89]. The finding underscores the importance of early HIV treatment for achieving optimal adherence in prisoners.

This study identified a significantly higher likelihood of viral non-suppression in males, people in the age group of 31 to 35 years and in those who encountered or perceived social stigma, irrespective of their incarceration status. Prior studies also showed higher odds of viral suppression in female prisoners than male prisoners [90]. With limited evidence available regarding the mechanism of how gender influences viral suppression, females often conform better to ART in the community settings [33, 59, 86], which might have also facilitated their adherence during incarceration.

Younger age (below 35 years) has been frequently reported to be associated with a higher risk of NA and viral non-suppression in both incarcerated [22, 27] and non-incarcerated populations [33, 59, 60]. People in this age group are generally more likely to adopt substance misuse behaviours and often encounter social stigma and discrimination [91]. Young adult males predominate prison populations in South Ethiopia [92] and around the world [93, 94], and they have a high prevalence of HIV infection compared to other age groups [95, 96]. Group specific HIV care intervention strategies including provision of adequate educational information about HIV and the importance of a consistent use of ART, are highly recommended.

The significant positive association between social stigma and viral non-suppression in this study may reflect the adverse effect of alienation on a patient’s appropriate use of medication [88, 97, 98], which is particularly profound in prison populations [17–19, 26]. Nonetheless, there existed no statistically significant association between social stigma and self-reported or pharmacy refill adherence in this study, which may represent a lower specificity of both methods in detecting adherence relative to plasma viral load measurement [41, 43, 99, 100]. Educational interventions are required to reduce this health related social stigmatisation by improving a general understanding of HIV amongst prison staff and prisoners [101].

This study had a few limitations. Approximately one quarter of correctional facilities present in South Ethiopia were included in the study based on the size of their prison populations. While there was no variation in treatment outcomes based on the type of correctional facility, it is still possible that ILWH who were in other prisons may have had different outcomes. A nationally representative study is required to draw conclusions that are illustrative of the prison populations in Ethiopia. Given the high turnover amongst prisoners and the high prevalence of sub-optimal ART outcomes in recidivists [102, 103], the prevalence of NA and viral non-suppression might have been underestimated in incarcerated people. Factors that affect ART outcomes throughout the incarceration cycle (during arrest, stay in jail, stay in prison and after release) should be longitudinally investigated by examining individuals at each stage of incarceration.

The participants’ true compliance to medication might have been over- or under-estimated as adherence in this study was measured using self-report and pharmacy refill methods [99, 100]. Self-reported adherence is likely to be threatened by recall and social desirability bias [100]. To minimise the effect of recall bias, short term (the previous four days) adherence was measured so that the participants’ memories about doses would be clearer. Strategies that could reduce the participants’ perceptions of the possible consequences of reporting adherence or non-adherence (such as reinforcing the importance of reporting both adherence and non-adherence for the research project, and reassurance that the information provided would not affect their care) were used to minimise social desirability bias. The pharmacy refill method of adherence measurement does not guarantee that clients could not obtain drugs from sources other than the reporting pharmacy, or provide information about when and how they take the medication [100]. Nonetheless, public health care facilities in the study area were almost exclusively providing ART services, which might have minimised an oversupply of drugs as only such institutions were involved in this study. In addition, when self-report and pharmacy refill methods are used in conjunction, the weakness of one approach could be offset by the strength of the other [100].

## Conclusions

This study revealed that sub-optimal dose adherence and viral suppression are generally higher in HIV-infected prisoners in South Ethiopia compared to their non-incarcerated counterparts. Structural, psychosocial, personal and clinical factors contributed to sub-optimal ART outcomes for prisoners. A discouraging institutional context hindered inmates from attending clinic visits, which increased the likelihood of dose-NA. While a lack of satisfaction with ART services predicted dose-NA in both incarcerated and non-incarcerated PLWH, prisoners were significantly less likely to be satisfied with ART services provided by external health care facilities. Experience of psychiatric distress and a lack of social support were found to be important psychosocial determinants of adherence in prisoners. Adherence to medication schedules, which itself was strongly influenced by the type of methods used to monitor time, predicted dose-adherence in both populations. Regardless of an incarceration status, males, people in the age group of 31 to 35 years and those who encountered social stigma were more likely to have viral non-suppression. The findings suggest a need for multilevel interventional approaches that focus on the specific needs of prisoners to alleviate these multiple barriers.

## Data Availability

The datasets used and/or analysed during the current study are available from the corresponding author on reasonable request.

## Abbreviations

ART: Antiretroviral therapy
ARV: Antiretroviral
AOR: Adjusted odds ratio
CD4: Cluster of differentiation-4
Chi^2^: Chi-square
CI: Confidence interval: COR: Crude odds ratio
DOT: Directed observed therapy
ILWH: Inmates living with HIV
MARPS: Most at risk groups
MCAR: Missing completely at random
MPR: Medication possession ratio
NA: Non-adherence
PAPI: Paper and pencil interviewing
PLWH: People living with HIV
RHB: Regional Health Bureau
RPHL: Regional Public Health Laboratory
SBREC: Social and Behavioural Research Ethics Committee
SNNPR: Southern Nations, Nationalities and People’s Region
SR: Self-report
SSA: Sub-Saharan Africa
TV: Television
UNAIDS: The Joint United Nations Programme on HIV/AIDS
USD: United States dollars
WHO: World Health Organization
